# The relationship between boredom proneness, the behavioral inhibition system, and anxiety in college students: variable-centered and person-centered analytic approaches

**DOI:** 10.3389/fpsyg.2024.1414736

**Published:** 2024-06-25

**Authors:** Mengmeng Zhao, Ruixin Wang, Zhenyu Zhao, Lina Li, Hongge Luo, Lei Wu

**Affiliations:** School of Psychology and Mental Health, North China University of Science and Technology, Tangshan, China

**Keywords:** boredom proneness, behavioral inhibition system, anxiety, college student, variable-centered and person-centered analytic approaches

## Abstract

**Objective:**

To explore the relationship among boredom proneness, the behavioral inhibition system, and anxiety among college students based on variable-centered and person-centered analytic approaches.

**Methods:**

A questionnaire survey was conducted on 1,102 college students from a university in Hebei Province using the Boredom Proneness Questionnaire (BPQ) for College Students, the Behavioral Inhibition System Scale (BIS), and the General Anxiety Disorder-7 scale (GAD-7).

**Results:**

The results reveal that boredom proneness was negatively correlated with scores on the behavioral inhibition system (*r* = −0.100, *p* < 0.01), and positively correlated with anxiety (*r* = 0.457, *p* < 0.001), while the behavioral inhibition system was positively correlated with anxiety (*r* = 0.086, *p* < 0.01). In the variable-centered analyses study, it was found that the behavioral inhibition system partially mediated the association between boredom proneness and anxiety. In the person-centered analyses study, three subtypes were identified: the high boredom-low inhibition group (9.35%), the moderate boredom-inhibition group (66.70%), and the low boredom-high inhibition group (23.95%). Individuals in these subtypes showed significant differences in anxiety scores (*F* = 4.538, *p* < 0.05), with the low boredom-high inhibition group scoring the highest.

**Conclusion:**

The results showed that the behavioral inhibition system partially mediates the relationship between boredom proneness and anxiety in college students; boredom proneness and the behavioral inhibition system exhibit group heterogeneity, with distinct classification features closely related to anxiety.

## Introduction

### Anxiety among college students

Anxiety disorders have emerged as one of the most pervasive mental disorders conditions globally, posing a substantial threat to the physical and psychological well-being of individuals (Liu et al., 2023b). Anxiety refers to the complex emotional state of tension and unease experienced by an individual when they perceive an impending or forthcoming event as threatening (Wang et al., 2018). Individuals afflicted with anxiety frequently manifest symptoms of heightened tension and trepidation, often accompanied by physical and emotional distress (Castro-Alija et al., 2023). Persistent feelings of anxiety constitute the primary etiological factor underlying the development of anxiety disorders, which can exert severe and protracted deleterious impacts upon the individual.

The college student population exhibits distinctive characteristics of anxiety in the process of psychological health development, manifesting in high-risk and dynamic trends. According to the “REPORT ON NATIONAL MENTAL HEALTH DEVELOPMENT IN CHINA (2021–2022),” an alarming 45.28% of college students are at risk of mild, moderate, or severe anxiety (Fu and Zhang, 2023). University students are in a critical period of identity formation, role adjustment, and psychological maturation (McDonagh et al., 2018). During this pivotal stage, they face numerous challenges, such as increased academic workload, complex interpersonal relationships, and uncertainties regarding career development. The presence of these factors has led to an increase in the incidence of anxiety and other mental health problems among college students (Jason et al., 2019; Yu et al., 2022; Cao, 2023). Furthermore, longitudinal empirical studies have corroborated the dynamic trajectory of college students’ anxiety (Liu et al., 2024). A study by Liu et al. (2023a) involving a four-year longitudinal survey of 2,473 college students found that the trajectories of anxiety can be categorized as the low and stable class, the increasing class, the increasing then decreasing class, the decreasing then stable class, and the high and decreasing class. This suggests that, influenced by various factors, college students’ anxiety is not a fixed and unchanging state. Furthermore, the high prevalence of anxiety among college students indicates that its impact cannot be overlooked. The continuously fluctuating nature of anxiety leaves individuals in a sensitive and fearful state, which not only increases their distress and psychological burden but may also lead to a range of adverse behaviors, such as intentional or unintentional self-harm, smoking, alcohol consumption, substance abuse, lack of physical exercise, and irregular sleep patterns (Tao et al., 2004; Alamoudi et al., 2023; Garit et al., 2023; John, 2023). Therefore, addressing anxiety among college students remains a significant and challenging issue.

### Boredom proneness and anxiety

Boredom proneness refers to the extent to which an individual tends to experience boredom or lacks stimulation, such as feelings of loneliness or a sense of meaninglessness, leading to a negative emotional state (Iannattone et al., 2024). While boredom proneness may appear subtle, it is closely associated with highly arousing negative emotional states, especially when individuals lack effective coping mechanisms for addressing boredom. According to Self-Regulation Theory, individuals achieve their goals by regulating their behavior, emotions, and cognition. When individuals experience a state of ennui or insufficient stimulation, they tend to adopt a detrimental attitude toward their own behavior and cognitive performance, resulting in deficiencies in self-regulation. This, in turn, leads to lazy behavior and negative emotion. Confronted with a task, they must strive to modulate their attention and emotions to accomplish it. However, boredom has already compromised their self-regulatory capabilities, rendering them ill-equipped to effectively manage the current work, thereby impeding their ability to derive satisfaction and fulfillment from their real-life experiences. Ultimately culminating in the emergence of negative emotions such as anxiety (Chen, 2016). Studies have empirically established that sustained experiences of boredom constitute a significant factor to the emergence of mental health concerns, including anxiety and depression, among college students (Miu et al., 2017). Therefore, boredom proneness represents an indispensable variable that must be accounted for when investigating the anxiety-related affective states of university populations. Based on this analysis, we propose:

Hypothesis 1: Boredom proneness positively predicts anxiety among college students.

### Boredom proneness, behavioral inhibition systems and anxiety

Reinforcement sensitivity primarily regulates organisms’ emotions and behaviors through the Behavioral Inhibition System (BIS) and the Behavioral Activation System (BAS). The Reinforcement Sensitivity Theory posits variations in sensitivity levels among individuals, leading to diverse experiences of stress, emotional distress, and responses to stimuli. Certain individuals exhibit heightened sensitivity to external factors and are more susceptible to environmental influences. The BIS, sensitive to punishing or threatening stimuli, induces passive avoidance and can trigger negative emotions, forming the basis for negative emotional responses (Gray, 1990; Carver and White, 1994; Liu et al., 2016). According to the susceptibility-stress model of anxiety, specific qualities, such as maladaptive personality traits and cognitive patterns, when activated by special environmental factors, heighten the risk of anxiety development (Mathews and MacLeod, 2005). The BIS can be regarded as a “vulnerability” factor, as individuals with elevated behavioral inhibition are more prone to adverse environmental effects, leading to negative developmental outcomes (Belsky and Pluess, 2009).

Previous studies have not correlated boredom proneness with the BIS, so the specific relationship between boredom proneness and behavioral inhibition is still unclear. However, when individuals experience boredom, BIS may be influenced. They may endeavor to take action to alleviate their state of ennui, as their craving for stimulation and activity is more acute. Consequently, these individuals may exhibit diminished behavioral inhibition in the process (Zuckerman and Neeb, 1979). Anxiety stems from goal conflicts, such as approach-avoidance or avoidance-avoidance, and is linked to passive avoidance behaviors (Neil, 1982). When the behavioral inhibition system activity is low in bored individuals, inhibiting impulses and seeking stimulation become challenging. These behavioral alterations focus attention and boost motivation, ultimately alleviating individual anxiety (Chao et al., 2023; Stone et al., 2023). Hence, we propose:

Hypothesis 2: Boredom proneness impacts individual anxiety through the mediating effect of the Behavioral Inhibition System.

### Variable-centered and person-centered analytic approaches

The variable-centered analyses primarily rely on group-level averages. However, for individuals, the notion of “average” often disregards the heterogeneity within the research sample, thereby diminishing the credibility of the research findings (Qiu, 2008). The person-centered analyses including Latent Class Analysis (LCA), Latent Profile Analysis (LPA), leverage the sample’s heterogeneity to determine the number of subgroups existing within a given sample, thereby reflecting the individual differences in the relationships between variables. This approach aligns more closely with the actual circumstances compared to traditional variable-centered methods (Pan et al., 2021). LPA inherits the statistical concept of the person-centered analyses from traditional latent class analysis, focusing on the heterogeneity among individuals. It not only provides a more precise delineation of the quantitative disparities between individuals but also summarizes the multidimensional qualitative distinctions among them (Su et al., 2024). Consequently, this study delves into the relationship between boredom proneness and anxiety in the BIS through a comprehensive analysis encompassing both variable-centered and person-centered perspectives.

### Research significance

This study adopted a multidimensional analytical approach to elucidate the intrinsic linkages among boredom proneness, BIS, and anxiety. While previous research has primarily focused on the influence of individual variables on anxiety, the current study comprehensively explored the complex relationships among the three variables from both variable-centered and person-centered perspectives, which contributes to a deeper understanding of the formation mechanism of anxiety in the college student population. The investigation not only considered the relationships among the variables, but also attended to individual differences, thereby generating more comprehensive research findings. Furthermore, the study examined the mediating role of the BIS in the relationship between boredom proneness and anxiety, and categorized the college student sample, providing new perspectives for subsequent research on the psychological underpinnings of anxiety in this demographic. These findings lay a theoretical foundation for the development of targeted intervention measures.

## Methods

### Participants

Determining an appropriate sample size is a critical step in research design. The commonly utilized formula for estimating the sample size required to assess a population proportion is as follows:


$$n=\frac{Z^{2}*p\left(1-p\right)}{e^{2}}$$

*n* represents the necessary sample size; Z denotes the critical value of the standard normal distribution, typically 1.96 for a 95% confidence level; p is the anticipated population proportion, often conservatively assumed to be 0.5 in the absence of prior information; e is the maximum allowable margin of error, commonly set at ±5% in research studies (Cochran, 1977). Applying this formula, the minimum required sample size was calculated to be 384.

The data collection was conducted on October 10, 2023. We collected 1,115 questionnaires from freshmen to senior students at a university in Hebei Province on the Questionnaire Star platform by using handy sampling. The present study employed rigorous screening protocols to ensure the quality and integrity of the collected survey data. Specifically, the following criteria were applied: (1) Completeness: Each questionnaire was meticulously examined to verify that all questions had been fully answered without any omissions. (2) Standardized Responses: Questionnaires exhibiting obvious repetitions or randomness in the responses were deemed invalid and excluded from the final analysis. (3) Consistency: Multiple responses from the same participant were cross validated to identify and remove any questionnaires with apparent contradictions or inconsistencies. Through this systematic screening process, a total of 1,102 valid questionnaires were retained, representing an effective response rate of 98.888%. 663 (60.1%) participants were female, and 439 (39.9%) were male, aged between 17 and 23 years (19.22 ± 1.75). All participants were college students, were free of mental illness and had signed informed consent forms.

### Measures

A sociodemographic questionnaire was used to collect information, such us sex and age. Additional instruments were used: Boredom Proneness Questionnaire (BPQ), Behavioral Inhibition and Activation Scale (BIS/BAS) and General Anxiety Disorder-7 (GAD-7) questionnaire. Participants were informed that the study would be conducted anonymously and that their data would not be disclosed.

The BPQ for College Students developed by Huang et al. (2010), comprises 30 items covering two dimensions: internal and external stimuli. External stimuli include four factors: constraint, monotony, loneliness, and tension, while internal stimuli consist of two factors: self-control and creativity. Employing a 7-point Likert scale (ranging from 1 for “completely disagree” to 7 for “completely agree”), with reverse scoring applied to the internal stimuli subscale, a higher mean score indicates a stronger tendency toward boredom. The Cronbach’s alpha coefficient for this scale is 0.9222 in this study.

The BIS and BAS developed by Carver and White (1994), drawing on Gray’s theory. The scale was revised by Li et al. (2008) in China, includes two subscales: the Behavioral Inhibition System (BIS) and the Behavioral Activation System (BAS). And BAS includes drive, pleasure-seeking, and reward responsiveness. The scale consists of 18 items, with this study concentrating exclusively on the Behavioral Inhibition subscale. Employing a 4-point Likert scale, each item was rated on a scale from “completely agree” to “completely disagree,” with scores ranging from 1 to 4. Higher scores indicate greater sensitivity. The Cronbach’s alpha coefficient for this scale in this study was 0.888.

The GAD-7 developed by Spitzer et al. (2006) and subsequently revised by Tong et al. (2016), which demonstrates good reliability and validity in screening for symptoms of anxiety. It consists of seven items, each rated on a four-point scale from 0 to 3. The total score is the primary statistical indicator, with higher scores indicating greater anxiety severity. Scores ranging from 0 to 4 indicate either the absence of anxiety or clinically insignificant levels, while scores from 5 to 9 denote mild anxiety, 10 to 14 indicate moderate anxiety, and scores of 15 or higher signify severe anxiety. The Cronbach’s alpha coefficient for this scale in this study was 0.965.

### Statistical analysis

The data was examined for common method biases using SPSS 23.0, employing tests such as Pearson correlation analysis, mediation effect test, and one-way analysis of variance. Additionally, LPA was conducted using Mplus 8.0.

## Results

### Common method bias

Utilizing Harman’s single-factor test approach, an unrotated exploratory factor analysis (EFA) was performed on all scale items within this study. The findings unveiled the presence of 6 factors with eigenvalues surpassing 1. The initial factor elucidated 31.195% of the variance (below 40%), suggesting the negligible presence of notable common method bias within this study (Zhou and Long, 2004).

### Correlation analysis

Pearson correlation analysis was utilized to examine the variables. The findings indicated a negative correlation between boredom proneness and the behavioral inhibition system (*r* = −0.100, *p* < 0.01), as well as a positive correlation between boredom proneness and anxiety (*r* = 0.457, *p* < 0.001). Additionally, a positive correlation was observed between the behavioral inhibition system and anxiety (*r* = 0.086, *p* < 0.01). This is illustrated in Table 1.

**Table 1:** Descriptive statistics and correlation analysis of variables (*r*).

Variable	*M ± SD*	BIS	BP	Anxiety
BIS	11.030 ± 2.868	1		
BP	104.298 ± 27.441	−0.100^**^	1	
Anxiety	4.990 ± 5.179	0.086^**^	0.457^***^	1

*N* = 1,102, ^***^p < 0.001, ^**^p < 0.01. BIS, behavioral inhibition system; BP, boredom proneness.

### Variable-centered analysis

Gender was included as a control variable and virtualized. Boredom proneness significantly and positively predicted anxiety (*β* = 0.089, *SE* = 0.005, *t* = 17.717, *p* < 0.001) and negatively predicted the behavioral inhibitory system (*β* = −0.010, *SE* = 0.003, *t* = −3.280, *p* = 0.001 < 0.01). Additionally, the behavioral inhibitory system significantly and positively predicted anxiety (*β* = 0.221, *SE* = 0.048, *t* < 4.559, *p* < 0.001), with a 95% confidence interval excluding 0. The results of the test for mediating effects are detailed in Table 2, demonstrating that the behavioral inhibition system partially mediates the relationship between boredom proneness and anxiety.

**Table 2:** Mediation effect of behavioral inhibitory system between the relationship boredom proneness and anxiety.

Pathway	Effect	Boost SE	95%*CI*	
			LLCI	ULCI
Total	0.087	0.005	0.077	0.097
BP → Anxiety	0.089	0.005	0.079	0.099
BP → BIS → Anxiety	−0.002	0.001	−0.004	−0.001

BIS, behavioral inhibition system; BP, boredom proneness.

### Person-centered analysis

**Figure 1:**
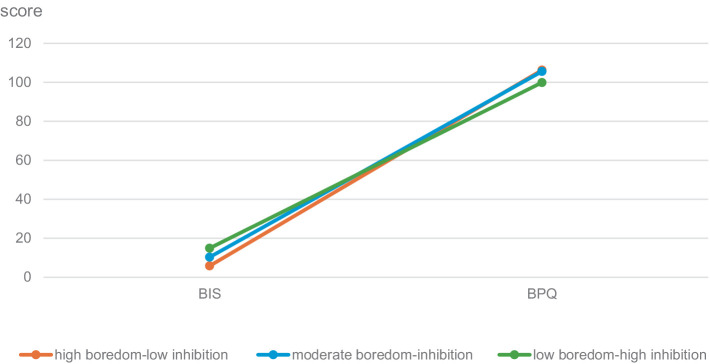
Characteristic graph about boredom proneness-behavioral inhibition system.

The boredom proneness-behavioral inhibition system served as the observational variable to construct 1–3 potential profile models sequentially, with the model fit indices presented in Table 3. According to the profile fit indices, smaller values of AIC and BIC denote better model fit, while an Entropy closer to 1 signifies more accurate classification (Wang et al., 2017). Significance results from the LMR and BLRT indicate a significant improvement in the model’s goodness-of-fit upon adding an additional profile. Thus, it can be concluded that this newly added profile is essential for explaining the data variability. Although all indicators of category 4 surpass those of category 3, a category within category 4 exhibits a smaller category probability, suggesting its lesser representativeness. Therefore, category 3 is deemed the most appropriate model. These categories were labeled based on their characteristics: the high boredom-low inhibition group, the moderate boredom-inhibition group, and the low boredom-high inhibition group (see Figure 1).

**Table 3:** Latent profile analysis model fit index.

Model	LL	FP	AIC	BIC	SSA-BIC	LMR(*p*)	BLRT(*p*)	Entropy	Categorical probability %
Class1	−7937.233	4	15882.466	15902.486	15889.781				100
Class2	−7906.055	7	15826.109	15861.144	15838.910	<0.001	<0.001	0.893	3.36/96.64
Class3	−7859.159	10	15738.318	15788.367	15756.604	0.049	<0.001	0.879	9.35/66.70/23.95
Class4	−7726.000	13	15478.000	15543.063	15501.772	<0.001	<0.001	0.942	62.80/9.35/2.99/24.86

### Effect of potential profile classification on anxiety

Table 4 shows the results. A one-way ANOVA was performed using boredom proneness-behavioral inhibition system subgroup classification as the independent variable and anxiety as the dependent variable. The results revealed a significant difference in anxiety scores among individuals with varying boredom proneness-behavioral inhibition system subgroup types (*F* = 4.54, *p* < 0.05). Specifically, the anxiety scores of the low boredom-high inhibition group were significantly higher than those of the moderate boredom-inhibition group, while the high boredom-low inhibition group did not differ significantly from the other two groups.

**Table 4:** Differences in anxiety by subgroup category.

Variable	Subgroup category scores on anxiety (M ± SD)			*F*	Back testing
	Class1 (103)	Class2 (735)	Class3 (264)		
Anxiety	5.250 ± 5.778	4.670 ± 4.656	5.770 ± 6.159	4.538^*^	2<3^*^

^*^p < 0.05, 1: the high boredom-low inhibition group, 2: the moderate boredom-inhibition group, 3: the low boredom-high inhibition group.

## Discussion

This study investigates the factors influencing anxiety from both the “variable-centered” and “person-centered” perspectives. The aim is to focus on the heterogeneity of individuals’ propensity for boredom proneness-behavioral inhibition system, and to explore the differences in anxiety levels among distinct types of individuals. The results of this study reveal the following: Firstly, the variable-centered research explored the mediating role of the behavioral inhibition system in the impact of boredom proneness on anxiety. Secondly, the person-centered research categorized the boredom proneness-behavioral inhibition system into three latent subgroups: the high boredom-low inhibition group, the moderate boredom-inhibition group, and the low boredom-high inhibition group, and discussed the impact of these different subgroup types on individual anxiety. The findings of this study validate the vulnerability-stress theory of anxiety.

### The effect of boredom proneness on anxiety among college students

The findings indicate that boredom proneness is a direct predictor of anxiety among college students, corroborating earlier research (Hym and Young, 2015) and supporting Hypothesis 1. On the one hand, the absence of adequate stimulation and goal-directed activities during boredom can result in unfocused attention and sluggish behavior. However, when faced with the pressure of completing tasks, the originally scattered attention of highly boredom proneness individuals may be forced to concentrate on the current task. Yet, due to their chronic self-regulatory deficits, these individuals struggle to adapt to such abrupt task shifts, potentially leading to an exacerbation of their anxiety levels (Westgate, 2020). On the other hand, the state of boredom can induce psychological stress stemming from a sense of wasted time or lack of achievement. This stress is likely to worsen upon the introduction of tasks, as individuals may become concerned about their capability to perform or meet standards, thus increasing the risk of emotional and behavioral dysregulation, as well as heightened anxiety and discomfort. Based on this, we inferred that boredom proneness directly predicts anxiety in college students.

The mediation analysis revealed that the behavioral inhibition system exerted a negative mediating influence on the relationship between boredom proneness and anxiety among college students. This suggests that by improving students’ boredom proneness, and thereby reducing behavioral inhibition, the aim of alleviating anxiety can be achieved. In other words, the BIS has a masking effect, partially offsetting the influence of boredom proneness on anxiety, thus verifying Hypothesis 2. Individuals with a high boredom proneness are particularly vulnerable to its emotional consequences, which can ensnare their thoughts in negative contexts. To escape this state of boredom, they are more likely to engage in impulsive or irrational actions, seeking immediate gratification or stimulation, while paying little attention to the consequences or long-term benefits. Such tendencies could lead to a diminished functioning of the behavioral inhibition system (Meng et al., 2017; Yan et al., 2021). Moreover, diminished activity in the behavioral inhibition system may correlate with a decrease in anxiety levels. Reduced activity suggests that individuals are more likely to initiate actions to manage stress and potential threats, thereby enhancing their perceived control over challenging situations and reducing the tension associated with anxiety. Conversely, high behavioral inhibition is more likely to exacerbate feelings of anxiety (Zhang et al., 2022; Tomé-Lourido et al., 2023).

### Classification of potential profiles of boredom proneness and behavioral inhibition system

Based on the variable-centered analysis, the study further delved into a person-centered analysis. The findings revealed that when categorizing the boredom proneness-behavioral inhibition system into three distinct latent groups, the fit indices were optimal. Specifically, the moderate boredom-inhibition group comprised 66.70% of the sample, the low boredom-high inhibition group constituted 23.95%, and the high boredom-low inhibition group comprised only 9.35%. Most individuals fell into the moderate boredom-inhibition group category, suggesting a prevalent state among most students. Attention and guidance are imperative to prevent these college students from transitioning into the low boredom-high inhibition category. Although the low boredom-high inhibition group represents a smaller proportion, their mental well-being necessitates scrutiny and intervention to mitigate anxiety stemming from boredom proneness and the behavioral inhibition system. Furthermore, these classifications offer fresh empirical evidence to deepen our understanding of predictive factors contributing to anxiety for college students.

### Effect of potential profile classification on anxiety

The impact of LPA on anxiety revealed significant differences in anxiety scores among individuals belonging to different subgroups of the boredom proneness-behavioral inhibition system. Specifically, the low boredom-high inhibition group exhibited significantly higher anxiety scores compared to the moderate boredom-inhibition group, while no significant difference was observed between the high boredom-low inhibition group and the other two groups. This finding aligns somewhat with the results of the mediation effect. Individuals with low boredom proneness and high behavioral inhibition activity demonstrated the highest anxiety levels. This may arise from the tendency of individuals with high behavioral inhibition to emphasize self-control and self-demand, thereby imposing greater pressure on themselves and experiencing difficulties in emotional regulation, ultimately leading to heightened anxiety (Afshadi et al., 2023). In contrast, individuals with moderate boredom-inhibition group displayed the lowest anxiety levels, possibly because moderate boredom fosters a more relaxed mindset, and appropriate behavioral inhibition enables individuals to confront pressure without resorting to avoidance or negative coping mechanisms, thereby facilitating the mitigation of negative emotions.

In summary, this study, employing both variable-centered and person-centered analyses and informed by self-regulation theory and susceptibility-stress model of anxiety, extensively investigated the relationship among boredom proneness, the behavioral inhibition system, and anxiety. The results from the variable-centered and person-centered analyses were corroborative. Boredom proneness positively predicts anxiety, yet the behavioral inhibition system acting as a regulatory mechanism counteracting the effects of boredom proneness on anxiety. The person-centered analysis revealed that individuals with moderate boredom-inhibition group exhibit the lowest levels of anxiety, while those with low boredom-high inhibition group experience the highest levels of anxiety. The findings reveal that the traditional variable-centered approach has produced contradictory results due to the neglect of sample heterogeneity. In contrast, the person-centered approach effectively addresses the limitations of the traditional method, providing a novel perspective on college students’ anxiety. This insight underscores the crucial role of individual differences in influencing variable relationships and highlights the importance of focusing on the potential differences among individuals (Zhou et al., 2024). Such an approach can aid in the identification of distinct population characteristics.

### Prospect

These results suggest that targeted interventions addressing both boredom proneness and the BIS may be effective in mitigating anxiety among college students. Firstly, personalized intervention approaches tailored to the unique characteristics of each subgroup could lead to more favorable outcomes. Secondly, by aligning interventions targeting the neural mechanisms of anxiety, such as transcranial magnetic stimulation (TMS) and neurofeedback training, with the distinct characteristics of different groups of college students, one can more effectively aid individuals in mitigating their anxiety. Finally, the integration of artificial intelligence technology presents novel opportunities to develop individualized anxiety management strategies based on students’ specific needs and profiles. Future research should explore the long-term efficacy of such personalized interventions and their potential to support the overall psychological well-being of diverse university student populations.

### Limitation

The limitations of the present study include the use of self-report scales, which are subject to subjectivity in responses. Additionally, cross-sectional design does not explain causal relationships. Future research could conduct 2–3 years of follow-up studies to better reveal the impact of boredom proneness on anxiety and other negative emotions from both variable-centered and person-centered analyses, to take effective intervention measures.

## Conclusion

First, the behavioral inhibition system partially mediates the association between boredom proneness and anxiety among college students. Second, boredom proneness and the behavioral inhibition system exhibit heterogeneity, comprising three distinct subgroups: the high boredom-low inhibition group, the moderate boredom-inhibition group, and the low boredom-high inhibition group. Finally, significant differences in anxiety levels are observed across these three subgroups, with the moderate boredom-inhibition group reporting the lowest scores, and the low boredom-high inhibition group exhibiting markedly higher anxiety levels in comparison to the moderate boredom-inhibition group.

## Data availability statement

The raw data supporting the conclusions of this article will be made available by the authors, without undue reservation.

## Ethics statement

Ethical review and approval was not required for the study on human participants in accordance with the local legislation and institutional requirements. The participants provided their written informed consent to participate in this study.

## Author contributions

MZ: Writing – review & editing, Writing – original draft, Investigation, Data curation. RW: Writing – review & editing, Investigation. ZZ: Writing – review & editing, Investigation. LL: Writing – review & editing, Supervision. HL: Writing – review & editing, Supervision. LW: Writing – original draft, Methodology.
